# Design Rules for Tuning Thermal Lifetimes of Arylazopyrazole Photoswitches

**DOI:** 10.1021/acscentsci.6c00586

**Published:** 2026-08-04

**Authors:** Maximilian X. Tiefenbacher, Johannes C. B. Dietschreit, Simon Axelrod, Rafael Gómez-Bombarelli, Leticia González

**Affiliations:** † Institute of Theoretical Chemistry, Faculty of Chemistry, 27258 University of Vienna, Währinger Straße 17, 1090 Vienna, Austria; ‡ Research Platform on Accelerating Photoreaction Discovery (ViRAPID), University of Vienna, Währinger Straße 17, 1090 Vienna, Austria; ¶ Department of Materials Science and Engeniering, Massachusetts Institute of Technology, 77 Massachusetts Avenue, Cambridge, Massachusetts 02139, United States; § Vienna Doctoral School in Chemistry, University of Vienna, Währinger Straße 42, 1090 Vienna, Austria

## Abstract

Predicting thermal relaxation rates in molecular photoswitches remains a major challenge, as competing reaction pathways and multiple electronic states govern their isomerization dynamics. Arylazopyrazoles exemplify this complexity while offering nearly quantitative bidirectional photoconversion and widely tunable thermal half-lives. Here, we map the isomerization landscapes of nearly 30,000 arylazopyrazole derivatives using a machine-learned interatomic potential trained on density functional theory data, enabling high-throughput simultaneous exploration of adiabatic and nonadiabatic pathways. The workflow identifies minima, transition states, and minimum-energy crossing points, allowing thermal relaxation rates to be computed at scale. Comparison with available experimental data demonstrates reliable prediction of absolute rate constants. Across chemical space, we uncover clear structure-mechanism-property relationships linking substituent position, pyrazole methylation, and electronic substitution patterns to mechanistic preference and overall relaxation kinetics. These results provide practical design rules for tuning the thermal stability of arylazopyrazole photoswitches and establish a general strategy for predicting relaxation kinetics in complex molecular switches.

## Graphical abstract

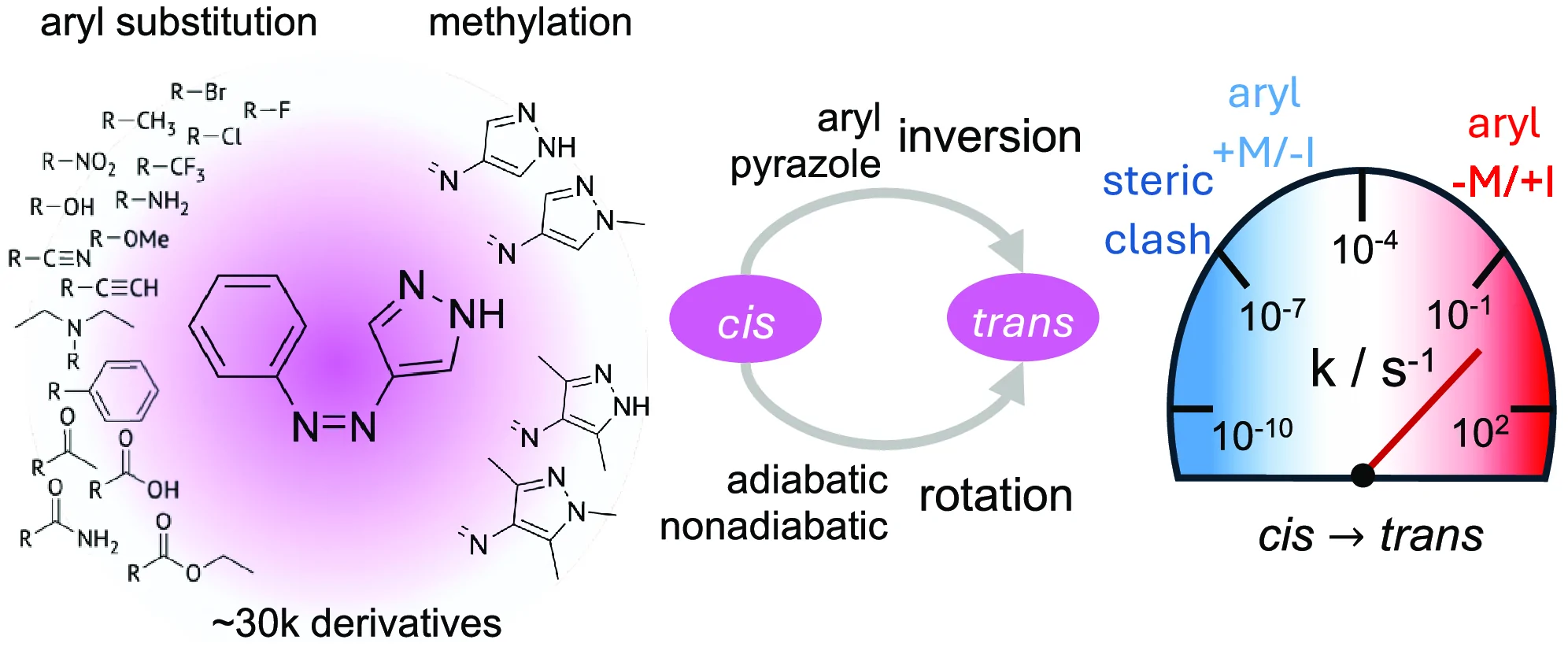

## Introduction

Photoswitchable molecules are a versatile class of functional compounds whose physical and chemical properties can be modulated with light.^1–3^ Their appeal has grown substantially in recent years due to a broad spectrum of applications, including organic electronics,^4,5^ energy storage,^6–9^ smart materials,^1,10^ data storage,^11,12^ and photopharmacology.^13–16^ In photopharmacology especially, the thermal half-life of the metastable isomer is a critical design parameter as it determines the duration of biological activity and must be tunable over many orders of magnitude to match the intended application. This motivates the search for molecules offering both reliable bidirectional switching and rationally adjustable thermal stability.^17^

Azobenzenes have long served as the prototypical scaffold in this field due to their robust *E*/*Z*-photoisomerization, high extinction coefficients, and excellent photostability.^18,19^ Yet classical azobenzenes suffer from two critical limitations: (i) incomplete photoswitching in both directions and (ii) relatively rapid thermal back isomerization, often occurring on the time scale of hours to days at room temperature.^20–22^ Numerous functionalization strategies have attempted to improve these properties,^23,24^ but establishing generalizable correlations between molecular structure, absorption behavior, and thermal half-lives remains challenging. A central reason for this difficulty is mechanistic: azobenzenes do not relax through a single, well-defined pathway. Different substitution patterns can give rise to distinct isomerization pathways, and reported mechanisms vary substantially among derivatives.^25,26^ This rich mechanistic isomerization landscape ranges from inversion of the bond angle on either side of the azo-bridge to rotation around the central N–N double bond, and may include any combination of these two mechanisms generally known as rotation-assisted inversion and inversion-assisted rotation.^27–29^ Additionally, rotational pathways can involve both singlet and triplet spin manifolds,^30^ with intersystem crossing (ISC) playing a more prominent role than initially thought.^31,32^ Although nonadiabatic rotational pathways were proposed nearly a century ago,^33^ appreciation of their broader relevance across chemical space has required the ability to compute barriers at scale, a capability that has only recently become practical.^32,34^

This variability among thermal relaxation pathways poses a fundamental obstacle to rational design: without knowing which pathway dominates for a given substitution pattern, it is almost impossible to predict how a structural modification will affect the thermal half-life. Limitations of azobenzenes for photopharmacology have prompted the search for alternative scaffolds capable of achieving both near-quantitative bidirectional switching and vastly extended thermal half-lives.^22,35^ Arylazopyrazoles (AAPs, structure shown in Figure 1) have emerged as highly promising next-generation photoswitches that meet these criteria. They provide almost complete photoconversion in both directions and exhibit thermal half-lives tunable over an exceptionally broad range—from minutes to several years—through appropriate substitution patterns.^25,26,36^ However, AAPs inherit many of the mechanistic complexities of azobenzenes: multiple thermal relaxation pathways, including competition between singlet and triplet surfaces.^37,38^ Critically, which of the different thermal pathways dominates is not a fixed property of the scaffold but shifts with substitution in ways that are not yet generalized.

**Figure 1:**
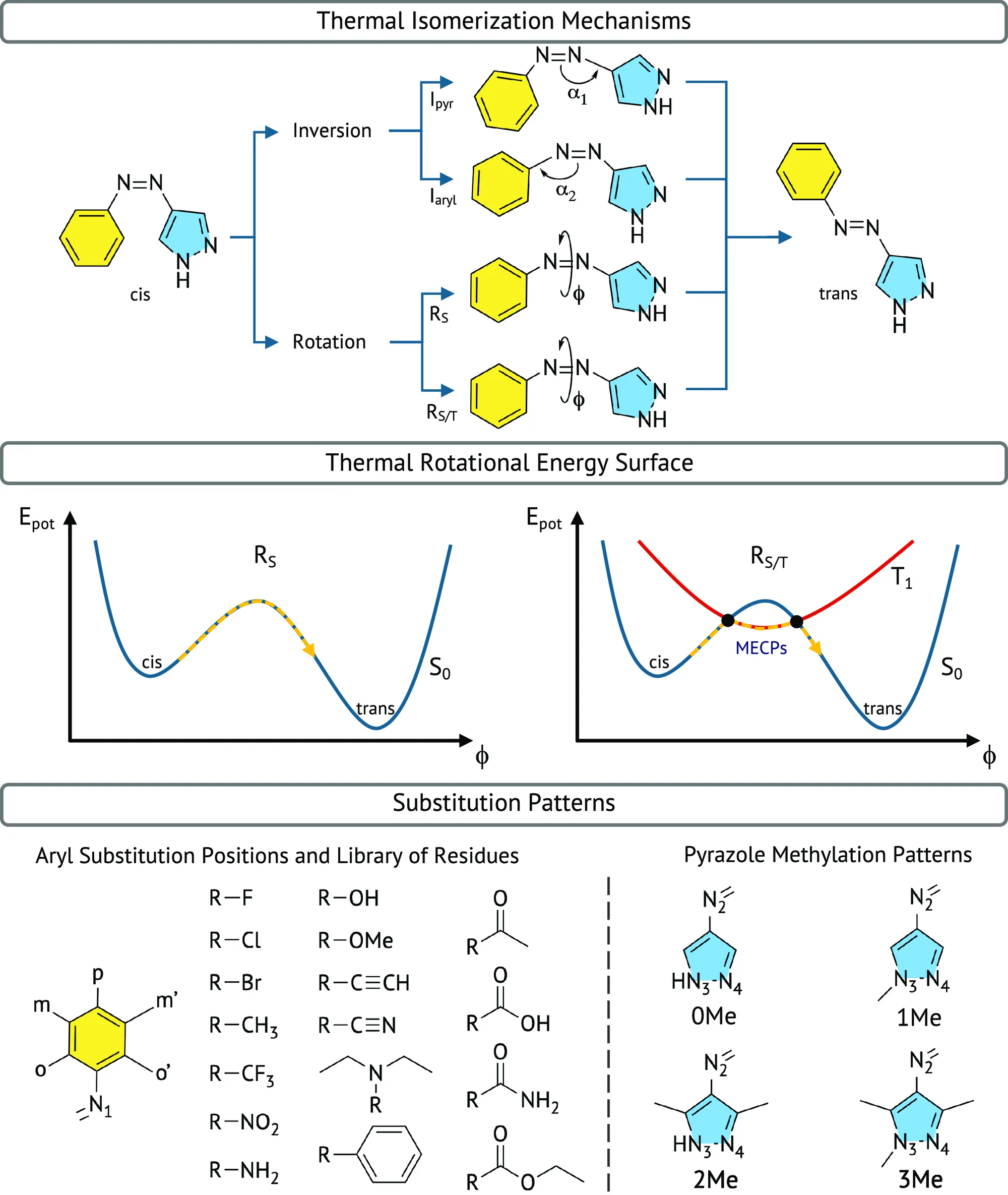
**Top**: *Cis*/*trans* thermal inversion and rotation isomerization pathways. Inversion can occur at either nitrogen atom, adjacent to the aryl (I_aryl_) or pyrazole (I_pyr_) ring, and is characterized by the CNN bending angle involving the azo nitrogens and the first carbon of the respective ring. Alternatively, isomerization may proceed via rotation around the N=N double bond, occurring either purely on the ground-state surface (R_S_) or via intersystem crossing to the lowest triplet state followed by a return to S_0_ (R_S/T_). The angles chiefly used to track the reactions are indicated for every pathway. **Middle**: Idealized depiction of the rotational potential energy profiles for the thermal ground-state isomerization mechanism R_S_ and the nonadiabatic mechanism R_S/T_ that involves the lowest triplet T_1_ (red surface). Minimum energy crossing points (MECP) are denoted by black circles. **Bottom**: Substitution patterns of arylazopyrazoles considered in this work. The aryl ring carries up to three of 17 substituents in the *ortho*-, *meta*-, or *para*-position relative to the azo bond. Four methylation patterns (zero to three methyl groups) were explored for the pyrazole ring.

Specifically, four different mechanisms (top of Figure 1) were identified through which the parent arylazopyrazole molecule can relax thermally from the *cis* to the *trans* minimum.^37^ They can be broadly classified as inversion (I) and rotation (R) pathways. The inversion can occur on either side of the azo-bridge, involving the pyrazole ring (I_pyr_) or the aryl ring (I_aryl_). The other two reaction mechanisms involve rotation around the central azo bond and differ in the electronic state(s) accessed during the process: the molecule might remain in the electronic singlet ground state (R_S_) or switch to the lowest lying triplet state (R_S/T_) as it rotates and then back before ending in the trans minimum (middle of Figure 1).^30^ The reaction path involving the triplet becomes important when the T_1_ energy is significantly lower than the S_0_ energy near the rotational barrier, and the spin–orbit coupling is strong enough to allow for population transfer between multiplicities.

Schlögl, Singer et al. showed that already one *para*-substituent on the aryl ring can heavily modulate the relative rates associated with the four different mechanisms, and the effect can be rationalized through Hammett parameters.^38^ However, for substitutions in *ortho*-position no such tabulated parameters exist, and the competition between simultaneously active pathways in polysubstituted derivatives remains largely unexplored. Resolving this interplay across a chemically diverse set of derivatives requires mapping all four reaction channels, including their transition states and intersystem crossing geometries, for thousands of compounds simultaneously. Any screening approach that collapses the problem to a single reaction pathway or ignores the triplet surface will misattribute the origin of observed rate variations and yield misleading design rules. High-level quantum chemical calculations can provide this mechanistic resolution for individual compounds,^39^ but their cost renders exhaustive exploration of even a modestly sized substituent library intractable.

To address the challenge of analyzing a large and diverse library of AAP derivatives, we turn to machine-learned interatomic potentials (MLIPs) that provide a cost-effective alternative to quantum-chemical simulations. MLIPs trained on high-level electronic-structure data can deliver good accuracy while enabling rapid evaluation of, e.g., energies and forces, across extensive chemical spaces.^40–44^ Importantly for photoswitching, modern MLIP architectures can represent multiple electronic states and their couplings, opening the door to automated exploration of complex isomerization landscapes at scale.^32^ Critically, the trained potential is deployed within a fully automated workflow that independently locates and verifies all four mechanistic pathways for each compound, and not simply the lowest-barrier route. This enables mechanistically resolved rate predictions across almost 30 thousand AAP synthetizable derivatives with mono-, di-, and trisubstitutions on the aryl ring coupled to four methylation patterns on the pyrazole ring (bottom of Figure 1).

By analyzing how different substitution patterns influence both overall relaxation rates and the competition among distinct pathways, we uncover generalizable trends within this chemical space. These insights culminate in a set of practical design guidelines that can support experimental efforts toward the rational development of AAPs with tailored thermal half-lives.

## Results and Discussion

### Generation of Derivatives

To systematically explore the chemical space of AAP derivatives, we generated SMILES strings covering all relevant substitution patterns on both the pyrazole and aryl rings, resulting in a library of 28,442 compounds.

For the pyrazole ring, we considered four distinct methylation motifs—denoted 0Me, 1Me, 2Me, and 3Me—corresponding to zero, one, two, or three methyl substituents, as illustrated in the bottom row of Figure 1. On the aryl ring, we employed a library of 17 substituents selected to span the relevant electronic (−M/+M, −I/+I) and steric range of the scaffold while remaining experimentally realistic.^25,36,45^ These substituents range from simple halogen atoms, over residues associated with typical push–pull effects to residues as large as phenyl rings. The set and position of the modifications was chosen in consultation with synthetic chemists (see Acknowledgments) in such a way that each derivative is reachable from commercially available or routinely prepared precursors through established azo-coupling and aryl-functionalization routes, without specialized methodology.

Each substituent can be placed at the *ortho*, *meta*, or *para* position relative to the azo bond, and we considered mono-, di-, and trisubstituted aryl patterns. For monosubstituted derivatives, the substituent on the aryl group can occupy the *ortho*, *meta*, or *para* position, giving rise to three distinct substitution patterns. For disubstituted derivatives, six unique positional arrangements are possible: *ortho*-*meta*, *ortho*-*para*, *ortho*-*meta*′, *ortho*-*ortho*′, *meta*-*meta*′, and *meta*-*para*, where the primed notation indicates substitution on the opposite side of the ring relative to the first substituent. For singly substituted derivatives, we do not use the prime notation, as these can simply interconvert by rotation around the single bond connecting the aryl ring to the azo-bridge. Finally, for trisubstituted derivatives, we selected two patterns based on synthetic feasibility and chemical relevance: *ortho-meta-ortho*′ and *ortho-para-ortho*′. All the aryl substitution patterns are paired with the four possible methylation patterns on the pyrazole ring. Together, these substitution patterns define the full set of structural motifs used to construct SMILES strings for all molecules included in this study.

### Rate-Constant Prediction Workflow

The automated workflow used to compute the quantities required for the rate expressions in classical (eq 1) and nonadiabatic (eq 7) transition-state theory is summarized in Figure 2 and described in detail in the Methods. As input, our code requires a SMILES string corresponding to the *cis*-isomer of the target molecule and the trained MLIP. The employed MLIP architecture is PaiNN and was selected because its fast equivariant message passing yields accurate forces and gradients. In addition, a shared molecular representation feeds separate output heads for the S_0_, S_1_, and T_1_ energies, providing the multistate description on which the nonadiabatic pathway relies. This architecture had already been validated for closely related excited-state photoswitch screening.^32,34^ Nevertheless, we expect that the structure–mechanism–property relationships reported here are not specific to this choice. The MLIP was trained on a diverse set of AAP derivatives with over 8,000 configurations. Reference energies and forces were computed using spin-flip time-dependent density functional theory (SF-TDDFT) at the BHHLYP/def2-SVP^46–48^ level of theory. Further details on data set generation and training are given in the Methods.

**Figure 2:**
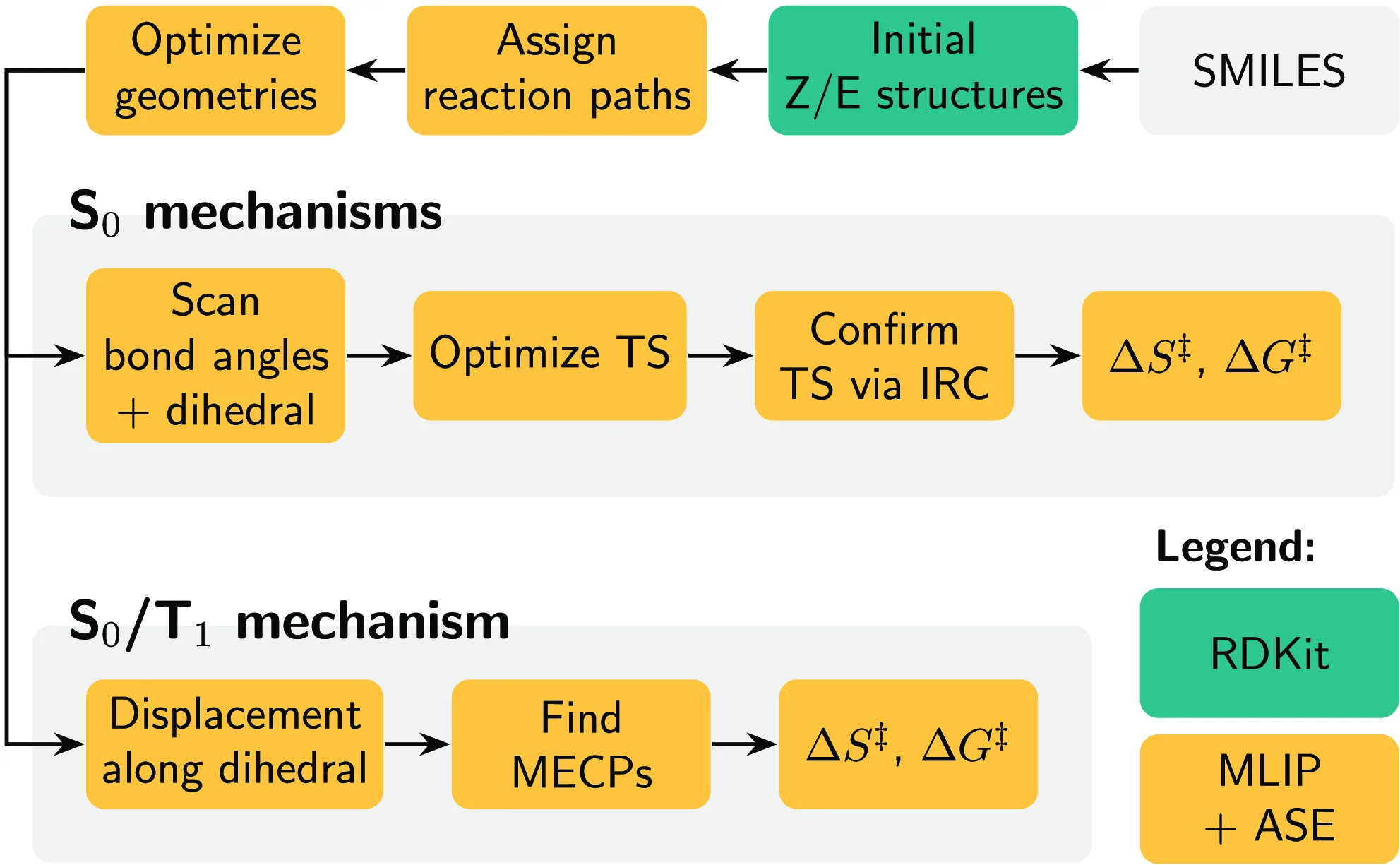
Schematic overview of the workflow used to compute the Gibbs free energies and corresponding rate constants for the four investigated isomerization mechanisms.

To compute the rate, we systematically generate initial conformers for every molecule, comprising the possible rotamers for each of the *cis* and *trans* isomers, yielding a total of six to eight structures per compound. After energy minimization, some of these collapse to a smaller number of distinct configurations. For locating the appropriate pairs of *cis* and *trans* minima, we inspect the angles formed by N_1_–N_2_–N_3_ and N_1_–N_2_–N_4_ (following the nitrogen numbering convention from Figure 1). During the rotational mechanism, the larger of these angles remains the larger one as the atoms move approximately on a cone around the azo-bond; during inversion, the order of the two angles reverses. This criterion allows us to assign each *cis* conformer with the correct *trans* conformer, depending on the pathway, which is required for identifying the minimum-energy crossing points (MECPs) between singlet and triplet surfaces for the R_S/T_-pathway. After locating all critical points and validating minima and transition states by frequency calculations, we compute the thermal corrections needed to compute Gibbs free energy barriers entering eq 1 and eq 7, for the adiabatic and nonadiabatic reaction mechanisms, respectively. More details can be found in the Methods.

### Machine-Learning Model Performance

We assess the performance of the MLIP-based workflow in Figure 3 by comparing predicted thermal relaxation rates with available experimental data for 15 para-substituted 3Me arylazopyrazoles at room temperature.^38^ Overall, the model reproduces absolute rate constants across more than 3 orders of magnitude. Specifically, nine of the 15 predicted rates lie within a single order of magnitude of the experimental value, and another one falls within 2 orders of magnitude. Given the exponential dependence of rate constant on the activation barrier, a deviation of 1 order of magnitude corresponds to an error of about 2.3 *k*_*B*_ *T* in the computed free energy barrier. This level of accuracy is well within what is expected for automated high-throughput TST workflows.

**Figure 3:**
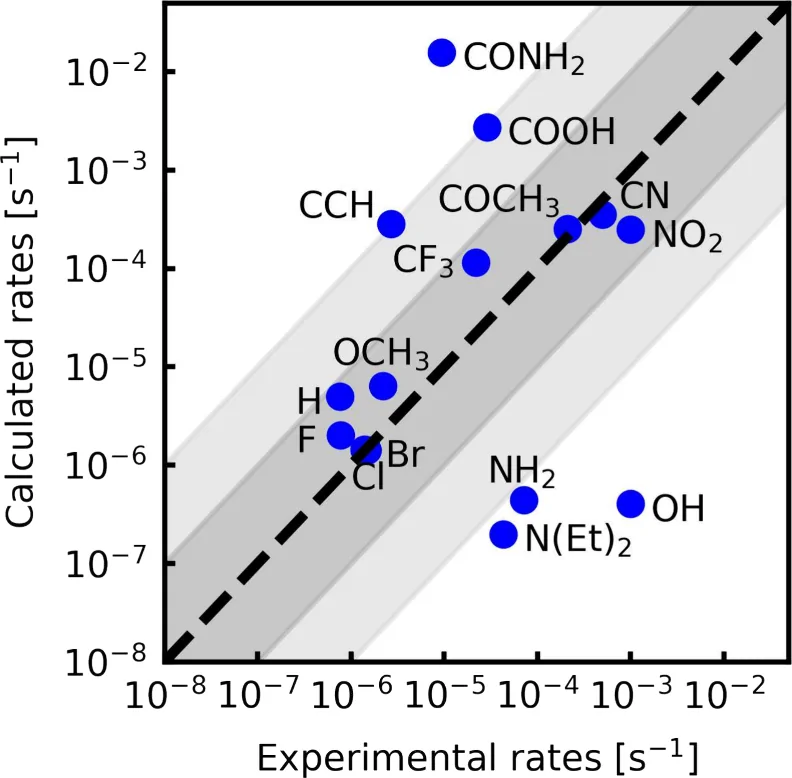
Comparison between experimental rates and predicted rates for 15 para-substituted 3Me derivatives from Schlögl, Singer et al.^38^ Both axes use logarithmic scales. The dark gray band marks deviations within a factor of 10; the light gray band corresponds to deviations within a factor of 1 hundred.

The remaining five cases show larger deviations, most of which can be traced to specific physical effects not captured by the current workflow. The largest deviations are associated with the substituents N­(Et_2_), OH, NH_2_ and CONH_2_, most of which can form hydrogen bonds with the solvent—effects that are not included in our automated approach. Capturing such interactions quantitatively would require explicit solvent, which removes the gas-phase rigid-rotor harmonic-oscillator approximation used throughout and replaces each barrier by a free-energy profile obtained through sampling. This would be incompatible with high-throughput screening. Some of these residues might additionally exist in multiple protonation states, further complicating any implicit solvation treatment. The last outlier is the ethynyl (CCH) substituent, which may exhibit electronic interactions that are not adequately captured by the MLIP, as most C–C bonds in the training set are single or double bonds. Further, the prediction for this compound also deviated significantly from the experimental value in a prior non-ML study,^38^ suggesting that the discrepancy originates, at least in part, from limitations of SF-TDDFT rather than the MLIP itself.

Together, these results establish that the machine-learned potential captures the relevant features of the ground- and excited-state energy landscapes, such that the ML-based workflow delivers quantitatively meaningful rate predictions with sufficient accuracy to support large-scale screening across thousands of molecules. However, before moving on, we want to point out that the comparison in Figure 3 is limited to *para*-monosubstituted 3Me derivatives, the only compounds for which experimental rates are available, and that the relative rates of a given substituent at this position do not transfer directly to other positions or to multiply substituted derivatives.

### Distribution of Relative Rates

Before examining how specific structural features tune the thermal relaxation rate, it is instructive to establish the overall landscape of rate constants across the full library and to understand how the four mechanistic pathways contribute individually and collectively. Figure 4a provides this overview, showing the distribution of predicted rate constants for all four isomerization pathways and their pairwise correlations across the full data set. Comparing mean values, aryl inversion (I_aryl_) is the fastest mechanism on average, followed by the two rotational pathways (R_S/T_ and R_S_), with pyrazole inversion (I_pyr_) exhibiting the slowest mean rate. However, mean values alone do not capture the full picture, and a closer inspection of the distributions reveals several important features.

**Figure 4:**
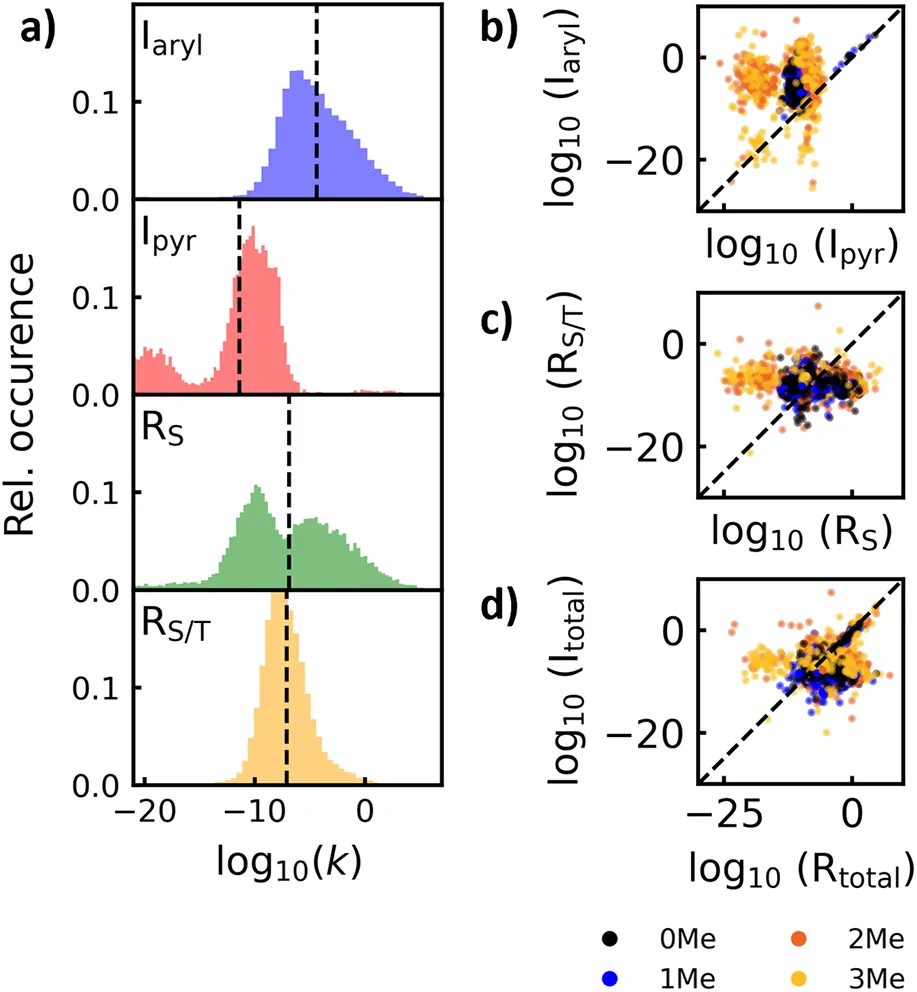
**Left**: (a) Relative distribution of log_10_(*k*) (with *k* in s^–1^) for all molecules, grouped by isomerization mechanism. From top to bottom: aryl inversion (I_aryl_), pyrazole inversion (I_pyr_), rotation about the N=N bond on the ground-state surface (R_S_), and rotation involving the lowest triplet state (R_S/T_). **Right**: Pairwise correlation of log_10_(*k*/s^–1^) between isomerization mechanisms. (b) Aryl inversion (I_aryl_) vs pyrazole inversion (I_pyr_). (c) Triplet-mediated rotation (R_S/T_) vs adiabatic ground-state rotation (R_S_). (d) Total inversion rates vs total of rotational rates. The color of each data point indicates the number of methyl substituents on the pyrazole ring (0Me–3Me), as defined in Figure 1.

The rate constants for ground-state rotation (R_S_) span an exceptionally broad range, covering many orders of magnitude, meaning this mechanism contributes to both some of the slowest and some of the fastest predicted isomerization rates across the library. This widespread reflects the strong sensitivity of the rotational barrier to both substituent pattern and pyrazole methylation. The distribution for pyrazole inversion (I_pyr_) is bimodal, with a secondary peak at very low rates, suggesting that for certain substitution motifs this pathway becomes strongly disfavored. In contrast, the triplet-mediated rotational mechanism (R_S/T_) displays a comparatively narrow rate distribution, indicating that the MECP barrier is relatively insensitive to substitution across chemical space. We note that the relative importance of the two rotational mechanisms may be subject to systematic uncertainty, since TDDFT has been shown to be insufficient for accurately describing the competition between adiabatic and nonadiabatic rotational pathways in related systems.^32^

Figure 4 panels b–d compare rate constants pairwise and reveal a pronounced dependence on pyrazole methylation across all panels: 0Me and 1Me molecules cluster relatively narrowly, whereas 2Me and 3Me derivatives span a substantially broader range, consistent with the greater steric complexity introduced by additional methyl groups.

Figure 4b shows the relationship between the two inversion pathways and makes the bimodal distribution of I_pyr_ clearly visible. All molecules in the slow-inversion regime belong to the 2Me or 3Me families, and a detailed inspection of the corresponding molecules reveals that these cases predominantly carry substituents at one or both *ortho* positions of the aryl ring. This indicates that steric repulsion between the pyrazole methyl groups and the *ortho* substituents hampers the out-of-plane motion required for pyrazole inversion, leading to strongly suppressed I_pyr_ rates.

Figure 4c compares the two rotational mechanisms. The nonadiabatic pathway R_S/T_ shows the narrow distribution already seen in the corresponding histogram of panel (a), reflecting a relatively uniform MECP barrier across chemical space. In contrast, R_S_ spans several orders of magnitude and displays the greatest variability among 2Me and 3Me derivatives, similar to the pyrazole inversion rates. This points to a stronger sensitivity of the ground-state rotational barrier to both steric and electronic substituent effects. It should be noted that the slowest ground-state rotation rates again occur for molecules with 2Me and 3Me methylation patterns, but also the fastest, completely encompassing single and double methylation patterns.

Figure 4d compares the total inversion rate with the total rotational rate for each molecule. Nearly all molecules exhibit overall rates above *k* = 10^–10^ s^–1^, confirming that even when one mechanism is strongly suppressed, at least one viable relaxation pathway remains accessible at room temperature—a direct consequence of the multichannel nature of arylazopyrazole isomerization. Notably, although I_aryl_ has the highest mean rate of the four individual pathways, as shown in Figure 4a, the majority of molecules nevertheless exhibits a larger total rotational rate than total inversion rate. This apparent reversal arises because the broad distribution of R_S_ pushes a large fraction of molecules into a rotation-dominated regime, even though the mean inversion rate is higher.

Figure 5 illustrates how the position of a single aryl substituent influences the total relaxation rate. Panel (a) compares *ortho*- and *meta*-substituted derivatives. Meta substitution produces a remarkably narrow rate distribution, with most molecules clustering between log_10_(*k*/s^–1^) = −6 and −4 and only weak dependence on pyrazole methylation. This insensitivity is consistent with the *meta* position being conjugatively decoupled from the azo linkage, so that substituents at this position exert neither a strong electronic nor a direct steric influence on the isomerization barrier. In contrast, *ortho*-substituted derivatives span nearly 10 orders of magnitude, underscoring the strong sensitivity of the *ortho* position, where substituents can simultaneously affect the electronic structure of the azo bridge and introduce steric strain, directly modulating the accessibility of both inversion and rotational pathways. Panel (b) compares *ortho*- and *para*-substituted derivatives. The spread in *para*-substituted rates closely mirrors that of the *ortho* series, confirming that *para* substitution exerts a comparably strong influence on the relaxation rate in contrast to substitutions in *meta* position. Since the *para* position is remote from the azo bridge, steric effects are negligible there, and the broad rate distribution must arise from electronic conjugation through the aryl ring. The similarity between *ortho* and *para* rates suggests that both positions participate in tuning the electronic environment of the azo-linkage, whereas *meta* substitution, being conjugatively decoupled, affects the mechanism only weakly. The additional steric involvement of the single *ortho* substitutions appears to play a minor role.

**Figure 5:**
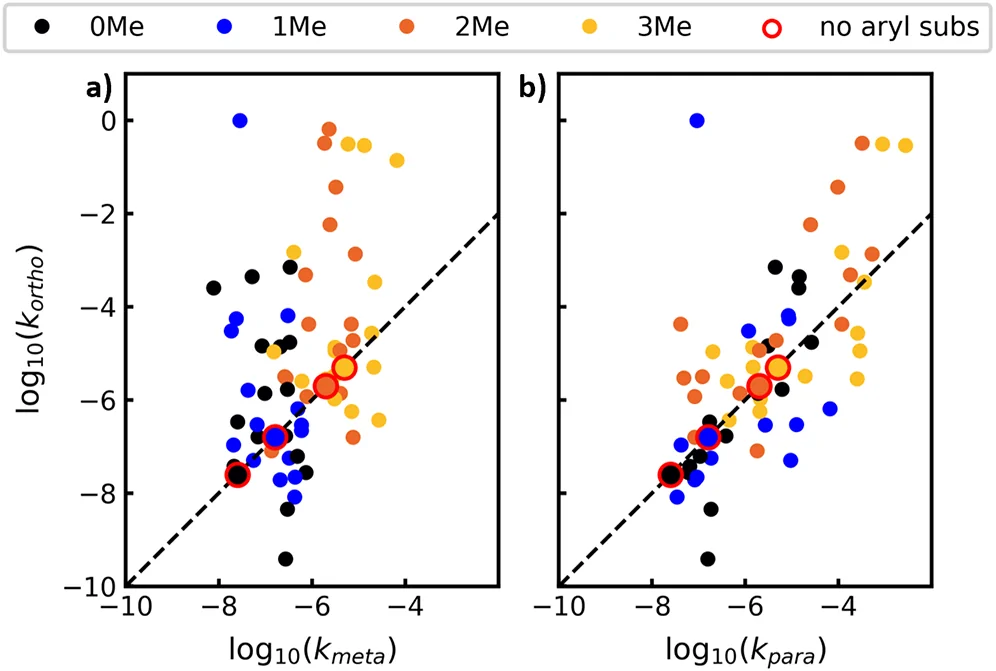
Comparison of total relaxation rates for aryl-monosubstituted AAP derivatives. (a) *Meta*-substituted rates versus *ortho*-substituted analogues. (b) *Para*-substituted rates versus *ortho*-substituted analogues. All rates are shown as log_10_(*k*/s^–1^). The color of each point indicates the number of methyl groups on the pyrazole ring (0Me–3Me), as defined in Figure 1.

Together, these observations establish a clear positional hierarchy: *ortho* and *para* substitution both strongly influence the total relaxation rate through electronic conjugation, with *ortho* substitution carrying the additional capacity for steric modulation, while *meta* substitution are comparably weak.

### Influence of Pyrazole Methylation Patterns

Having established the overall rate distributions, we now turn to identifying which structural features are responsible for pushing molecules into the fast- or slow-relaxing extremes of the distribution. The first column of Figure 6 addresses this by comparing the relative occurrence of structural elements among the fastest and slowest compounds in the library, where we classify rates as fast if log_10_ *k* > $\overset{®}{\log_{10}k\;}$ + σ­(log_10_ *k*) ≈ −2.6 and as slow if log_10_ *k* < $\overset{®}{\log_{10}k\;}$ – σ­(log_10_ *k*) ≈ −7, with $\overset{®}{\log_{10}k\;}$ and σ­(log_10_ *k*) denoting the mean and standard deviation of the logarithmic rate constants across the full library.

**Figure 6:**
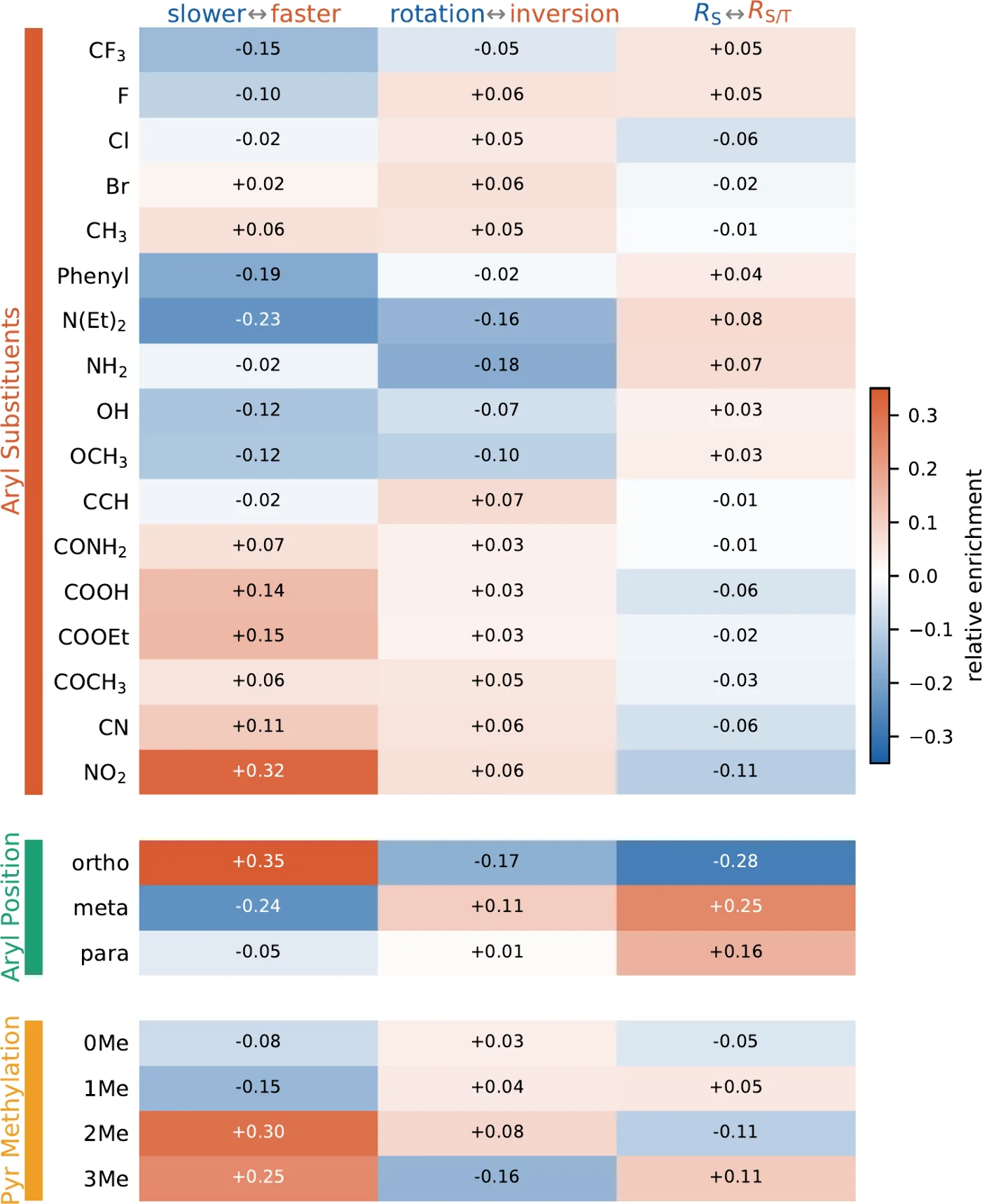
Relative enrichment of substitution patterns across three mechanistic descriptors of arylazopyrazole (AAP) photoswitch relaxation: overall relaxation rate (slower vs faster), relaxation mechanism (rotation vs inversion), and rotational pathway (R_S_ vs R_S/T_). Rows correspond to individual substitution features, grouped by category (color bar, left): aryl-ring substituent identity, substitution position on the ring, and pyrazole methylation pattern. Cell color encodes the relative enrichment score; coral indicates enrichment toward the first-named pole of each descriptor (faster relaxation, inversion mechanism, triplet-mediated rotation), blue toward the second (slower relaxation, rotation mechanism, ground-state rotation). Color scale is shared across all three columns to allow direct comparison of enrichment magnitude between descriptors.

Inspection of the bottom panel of Figure 6 reveals a clear dependence of the thermal relaxation rate on the pyrazole methylation pattern. Derivatives in which the pyrazole carbon atoms adjacent to the azo bond are methylated (2Me and 3Me) are significantly overrepresented among the fast-relaxing compounds, suggesting that methyl groups at these positions destabilize the *cis* minimum or lower the isomerization barrier through steric pressure near the azo bond. In contrast, structures lacking methyl groups at these positions (0Me and 1Me) are more frequently associated with slower relaxation, although this effect is less pronounced than the enrichment of 2Me and 3Me derivatives among fast-relaxing species.

A mechanistically more detailed picture emerges when we recall Figure 4b, which shows that for the pyrazole inversion pathway, the 2Me and 3Me derivatives split into two distinct clusters: the majority exhibit inversion rates comparable to 0Me and 1Me molecules (centered around log_10_ (*k*/s^–1^) ≈ −10), while a subset forms a separate cluster at much lower rates around log_10_ (*k*/s^–1^) ≈ −20. This bimodality indicates that although pyrazole methylation generally enables isomerization by increasing steric pressure near the azo bond, specific structural combinations can strongly suppress the inversion pathway instead. A similar but less pronounced clustering is observed for the adiabatic rotational mechanism in Figure 4c, where a group of highly methylated AAPs exhibits the slowest R_S_ rates. When inversion and rotation contributions are summed in panel (d), only a small number of molecules—almost exclusively 3Me derivatives—remain in the slowly rotating cluster.

Structural analysis of these slow-relaxing outliers reveals a common motif: one or both *ortho* positions of the aryl ring are occupied by sterically demanding groups such as phenyl or N­(Et)_2_. The combination of bulky *ortho* substituents on the aryl ring with extensive pyrazole methylation appears to introduce severe steric congestion, inhibiting both inversion and rotational pathways and leading to exceptionally long thermal half-lives. In summary, these findings highlight pyrazole methylation as a powerful, but very context-dependent lever for tuning thermal relaxation rates. While methylation generally accelerates isomerization, its interplay with aryl substitution enables access to both fast- and slow-relaxing regimes.

### Influence of Aryl Substitutents

We now ask how the chemical identity and electronic character of the aryl substituents determine whether a compound falls into the fast- or slow-relaxing regime. The top and middle panels of Figure 6 address this directly by comparing substituent occurrence and position between the two extremes of the rate distribution. The following design rules are derived from population-level enrichment across the full library rather than from individual absolute rates, so that per-compound deviations do not affect qualitative trends.

Among fast-relaxing compounds, the most strongly enriched substituents are those with a mesomeric electron-withdrawing character (−M): NO_2_ shows the highest enrichment, followed by COOH and COOEt at comparable levels, then CN, and finally COMe and CONH_2_. It is likely that these substituents destabilize the *cis* relative to the *trans* isomer and lower the activation barriers for both inversion and rotation through electron withdrawal from the azo bridge, thereby accelerating relaxation. Methyl groups, which exert a weak electron-donating inductive effect (+I), show a modest but consistent enrichment among fast compounds at a level comparable to COMe and CONH_2_.

The slow-relaxing compounds display a complementary electronic profile. Phenyl and N­(Et)_2_ groups are the most prominent among them, followed by CF_3_, F, OH, and OMe. These substituents fall into two distinct electronic categories: those with an electron-withdrawing inductive effect (−I) such as CF_3_ and F, and those with a mesomeric electron-donating character (+M) such as OH, OMe, and N­(Et)_2_. The enrichment of both −I and +M substituents among slowly relaxing AAPs points to two electronically distinct routes to kinetic stabilization of the *cis* isomer, despite their opposing electronic characters.

The substitution position reinforces these trends. Nearly all fast-relaxing compounds carry at least one *ortho* substituent, a strong enrichment that is absent for *meta* substitutions. This indicates that *ortho* groups play an active role in destabilizing the *cis* geometry or facilitating access to the transition state, most likely through a combination of steric distortion of the azopyrazole framework and direct electronic conjugation with the azo bridge—both effects being unavailable to *meta* substituents, which are conjugatively decoupled from the reaction center.

Together, these findings define a clear structure–rate relationship for AAP derivatives. Rapid thermal relaxation is promoted by −M or +I aryl substituents, *ortho* substitution, and higher pyrazole methylation, while slow relaxation is favored by +M or −I substituents combined with low methylation. The one important exception to this electronic picture is that bulky *ortho* substituents paired with high pyrazole methylation can override the normally rate-accelerating effect of *ortho* substitution, creating exceptionally slow-relaxing compounds through steric congestion alone. These principles provide an experimentally useful framework for designing AAP photoswitches with prescribed thermal half-lives.

### Change in Mechanism

In addition to investigating which substitution patterns lead to an overall slow or fast thermal relaxation, we computed the enrichment of aryl substituents and pyrazole methylation patterns for the cases in which the overall rotation rate is larger (or smaller) than the inversion rate, as well as for the cases in which triplet-mediated rotation (R_S/T_) is favored over ground-state rotation (R_S_). The results are shown in the second and thurd column of Figure 6. This enrichment analysis was carried out irrespective of whether the compounds were overall fast or slowly relaxing, simply by comparing the respective rate constants.

We find that the enrichment is not as clear-cut as for the overall relaxation rate. However, a similar electronic picture emerges. The presence of electron-donating groups (+M) appears to favor inversion, whereas electron-withdrawing groups (-M) have the opposite effect. Similarly, electron-donating groups favor triplet-mediated rotation over a purely singlet, ground-state rotation mechanism, while the opposite holds for electron-withdrawing (−M) groups. This is in excellent agreement with the findings of Schlögl et al.,^38^ who investigated singly-*para*-substituted AAP compounds and found that substituents with a negative Hammett parameter (i.e., electron-donating) enable triplet-mediated rotation, whereas substituents with a positive Hammett parameter favor aryl inversion.

This picture also fits with the enrichment of substitution positions. On the aryl ring, *ortho*-substitution is enriched in compounds with a dominant rotation mechanism. In general, aryl inversion is faster than pyrazole inversion, and substituents in the *ortho* position simply hinder (aryl) inversion, thereby promoting rotation. A very strong *ortho*-enrichment is also found for compounds where *k*_R_S__ > *k*_R_S/T__; in contrast, *meta*- and *para*-substitution are enriched for compounds where *k*_R_S__ < *k*_R_S/T__. Again, this fits with the previous findings of Schlögl et al.,^38^ where triplet-mediated rotation outweighed the ground-state alternative pathway for every molecule studied. The R_S_ mechanism is only dominant in compounds bearing strongly electron-withdrawing groups in the *ortho* position, where they have the strongest influence on the double bond, destabilizing the triplet with respect to the ground state, raising the energy of the MECPs and thus decreasing *k*_R_S/T__.

## Conclusions

In this work, we have shown that machine-learned interatomic potentials trained on spin-flip TDDFT data can be deployed within a fully automated workflow to predict thermal relaxation rates of arylazopyrazole photoswitches with near-*ab initio* accuracy and at a throughput that makes exhaustive chemical space exploration tractable. Importantly, the workflow resolves all four competing isomerization mechanisms—generally denominated as aryl inversion, pyrazole inversion, adiabatic ground-state rotation, and nonadiabatic triplet-mediated rotation—for each compound independently. This mechanistic resolution is essential as the dominant pathway shifts with substitution pattern. Case in point, each of these mechanisms has been invoked in mechanistic experimental and theoretical studies of azo-based photoswitches.^28,30–32,38^

Applying our automated methodology to almost 30 thousand arylazopyrazole derivatives reveals that thermal stability in arylazopyrazoles is governed by a small number of cooperating and competing structural principles. Electronic effects at the ortho and para positions tune the azo bridge through conjugation, with −M substituents accelerating and +M or −I substituents retarding relaxation. Pyrazole methylation generally promotes faster isomerization, but its interplay with aryl substitution is not additive: the combination of extensive methylation with bulky ortho groups introduces steric congestion that can override the electronic trends entirely. The meta position, being conjugatively decoupled from the reaction center, contributes neither strong electronic nor steric modulation.

In conclusion, our work demonstrates that arylazopyrazole thermal stability can be tuned over many orders of magnitude by cleverly combining electronic and steric effects. Our findings reduce a complex, multipathway problem to a compact set of actionable design rules: fast-relaxing compounds are accessed by combining −M aryl substituents in *ortho* or *para* position and high pyrazole methylation, while slow-relaxing compounds are favored by +M or −I substituents, low methylation, or the deliberate introduction of steric congestion. With all predicted rates, associated structures, workflow scripts, and trained potentials available on Zenodo, this work provides both a validated screening resource and a mechanistic foundation for the rational design of arylazopyrazole photoswitches.

Finally, while the trained potential reported here is specific to the arylazopyrazole chemical space and would require retraining for chemically distinct scaffolds, the workflow itself is scaffold-agnostic, and therefore transfers directly to other photoswitches built around an azo-linkage, and with some modifications also to those built around a C–C double bond. Like the workflow, we expect the central design principles to transfer beyond arylazopyrazoles to other photoswitches that isomerize about a central double bond. The stereoelectronic effects—–M substituents weakening the azo π system and thereby lowering the rotational barrier, and the steric destabilization of the *cis* isomer that accelerates relaxation until overcongestion instead traps it—follow general chemical reasoning and should apply to azobenzenes and arylazopyridines/pyrimidines, and, for the rotational and steric channels only, to stilbene-type switches that lack the azo lone pairs and hence the inversion pathways. Scaffold-specific, by contrast, are the features tied to the pyrazole ring as well as the relative ordering of the four channels, and the prominence of the triplet pathway, all of which depend on the specific heteroaryl and its S_0_/T_1_ energetics.

## Methods

### Transition-State Theory

Since the thermal relaxation of arylazopyrazoles may proceed either entirely on the singlet potential energy surface (PES) or through a sequence of transitions involving both singlet and triplet states, the corresponding rate expressions depend on whether the reaction follows an adiabatic pathway or a nonadiabatic one that requires ISC.

If only the ground-state singlet PES is involved, the relaxation rate can be computed using conventional transition-state theory (TST).^49–51^ In this case, the rate constant *k* is given by

1 $$k=\frac{k_{B}T}{h}\;\exp\left(-\frac{\Delta G^{‡}}{k_{B}T}\right)$$

where *k*_*B*_ is Boltzmann’s constant, *T* is the absolute temperature, *h* is Planck’s constant, and *ΔG* ^‡^ is the Gibbs activation free energy associated with the *cis/trans* isomerization. In the present context, *ΔG* ^‡^ corresponds to the free energy difference between the *cis* minimum and the singlet transition state.

When the triplet PES participates in the relaxation mechanism, the overall rate is governed not only by the activation free energies but also by the probability of switching between electronic states. To describe this process, we employ nonadiabatic transition-state theory (NA-TST),^31^ in which the efficiency of crossing between singlet and triplet surfaces is encoded in a transmission coefficient ϵ.^31,52^ The transmission coefficient for an ISC event is given by

2 $$\epsilon =\frac{\pi^{3/2}\eta }{2\sqrt{\lambda k_{B}T}}\left[1+\frac{1}{2}\;\exp\left(\frac{1}{12\eta^{2}{\left(\lambda k_{B}T\right)}^{3}}\right)\right]$$

where the parameters η and λ characterize the strength of the spin–orbit coupling (SOC) and the relative curvature of the two PESs near the crossing point:

3 $$\eta =\frac{4{H_{\mathrm{SO}}}^{3/2}}{h/2\pi }{\left(\frac{\mu }{F_{g}\left|\Delta F\right|}\right)}^{1/2}$$

4 $$\lambda =\frac{\left|\Delta F\right|}{2F_{g}H_{\mathrm{SO}}}$$

Here, *H*_SO_ encodes the magnitude of the spin orbit coupling vector between the singlet and triplet states at the crossing point, Δ**F** is the difference in the force vectors on the singlet and triplet surfaces, and *F*_*g*_ is given as

5 $$F_{g}={\left|\overset{N}{\underset{n=1}{\sum }}\overset{3}{\underset{j=1}{\sum }}F_{S,\mathrm{nj}}F_{T,\mathrm{nj}}\right|}^{1/2}$$

The reduced mass μ associated with the reaction coordinate is computed from the force difference vector,

6 $$\mu ={\left[\frac{1}{|\Delta F{|}^{2}}\overset{N}{\underset{n=1}{\sum }}\overset{3}{\underset{j=1}{\sum }}\Delta {F_{nj}}^{2}{m_{n}}^{-1}\right]}^{-1}$$

with *m*_*n*_ being the atomic masses.

In general, the relaxation mechanism may involve two distinct singlet–triplet crossing points (to switch from singlet to triplet surface and back, see second row of Figure 1), each characterized by a transmission coefficient (ϵ_1_, ϵ_2_) and an activation free energy (*ΔG*_1_^‡^, *ΔG*_2_^‡^) with respect to the *cis*-minimum. Following NA-TST, the overall nonadiabatic rate constant is then

7 $$k_{\mathrm{NA}‐\mathrm{TST}}\left(T\right)=\frac{k_{B}T}{h}\frac{\epsilon_{1}\;\exp\left(-\frac{\Delta G_{1}^{‡}}{k_{B}T}\right)\epsilon_{2}\;\exp\left(-\frac{\Delta G_{2}^{‡}}{k_{B}T}\right)}{\epsilon_{1}\;\exp\left(-\frac{\Delta G_{1}^{‡}}{k_{B}T}\right)+\epsilon_{2}\;\exp\left(-\frac{\Delta G_{2}^{‡}}{k_{B}T}\right)}$$

Equation 7 describes only the nonadiabatic mechanism and accounts for nonadiabatic branching and for the possibility that one crossing point dominates the relaxation at a given temperature.

To describe all rates together, eqs 1 and 7 are used in conjunction: the adiabatic rate expression (eq 1) is applied to the three singlet-surface mechanisms (I_aryl_, I_pyr_, and R_S_), while the nonadiabatic expression (eq 7) is applied to the triplet-mediated rotational channel (R_S/T_). The total rate is computed as

8 $$k_{\mathrm{tot}}=k_{R_{S}}+k_{R_{S/T}}+k_{I_{pyr}}+k_{I_{aryl}}$$

The total rate constant thus provides a description of the thermal relaxation, irrespective of whether the underlying mechanism is purely adiabatic, purely nonadiabatic, or a mixture of both.

### Training Set Generation

The training set consisted of all molecules containing zero or one substituent on the aryl ring, along with 1500 randomly selected di- and trisubstituted derivatives. Throughout the generation of this data set, xTB with the GFN2 semiempirical method^53^ was employed to perform rapid structure optimizations and to generate initial geometries along relevant reaction coordinates. Reference energies and forces for training the machine-learned potential were computed using spin-flip time-dependent density functional theory (SF-TDDFT) at the BHHLYP/def2-SVP level of theory,^46–48^ as implemented in Q-Chem version 5.4.^54^ SF-TDDFT was selected because it provides a balanced description of the near-degenerate S_0_/S_1_ states at the twisted rotational transition states and degenerate S_0_/T_1_ states at the singlet–triplet crossing points central to this study, where standard TDDFT is unreliable and to be consistent with prior machine-learning work on azobenzene thermal half-lives (refs 32, 34 .). Its principal limitation is spin contamination of the target states, which we address by discarding geometries with anomalous ⟨*Ŝ* ^2^⟩ (see details below); mixed-reference SF-TDDFT,^55,56^ which is largely free of spin contamination, was not available in the code used here but represents a promising direction for future higher-fidelity reference data.

During data generation, for each selected SMILES, both the *cis* and *trans* minima were optimized at this level. To sample the isomerization pathways, relaxed scans were performed along the inversion and rotational reaction coordinates, using 20 points connecting the *cis* and *trans* minima. In addition, eigenvector-following optimizations were conducted to locate approximate transition-state-like geometries. This procedure was applied to all monosubstituted molecules and to a random subset of 500 di- and trisubstituted derivatives. For every geometry generated from these displacement and optimization procedures, energies and gradients were computed for the S_0_, S_1_, and T_1_ states. Starting from this data set, three neural network potentials were trained with different initial weights. We used the PaiNN^57^ architecture with a feature vector length of 256, 3 convolutional layers and the default activation function “swish”. Each network has three output nodes, one for each of the S_0_, S_1_ and T_1_ energies, to which we add the D3 dispersion correction from Grimme^58^ to account for long-range interactions. We did not employ a pretrained foundational MLIP because current models are trained almost exclusively on ground-state energies and forces and do not provide the excited-state (S_1_, T_1_) surfaces we require. Fine-tuning a pretrained backbone on excited-state references is nonetheless an attractive route to lower data requirements, which might be pursued in future work.

Training was started with a learning rate of 10^–4^, which is decreased by a factor of 0.5 whenever the validation loss does not improve over 25 consecutive epochs. The training was stopped when the learning rate fell under 10^–7^. Otherwise, the maximum number of training epochs was 1000. The loss function is the sum of the mean absolute errors of all three target energies and their respective Cartesian gradients (negative forces), with gradients and energies having weights 5:1.

To improve the training set, we randomly selected one model to carry out the production workflow, as depicted in Figure 2 (excluding the triplet-mediated mechanism in this stage), for the subset of training molecules. For the obtained structures, energies and gradients from all three models were evaluated to gauge the disagreement among the three MLIPs as a diagnostic for prediction quality. The 2500 geometries exhibiting the largest discrepancies were selected for computation with the quantum-chemical reference method and incorporated into the training set. To avoid oversampling high-disagreement regions, additional 1000 randomly selected structures with low disagreement were also added. This active learning procedure was performed twice, with the first cycle contributing 3474 new structures and the second adding an additional 2405 structures. We discarded data points, which have a ${Ŝ}^{2}$ expectation of over 0.5 for singlet states or less than 1.6 for the triplet state, as calculations with a high degree of spin contamination (i.e., a large difference between computed and correct magnitude of $\left\langle {Ŝ}^{2}\right\rangle$ for a certain spin multiplicity) deviate strongly from the correct PES.

After the second active learning cycle, the average root mean squared error for the test set was reduced to 0.96 kcal/mol for the S_0_ state and 3.36 kcal/mol for the T_1_ state, which we consider sufficiently accurate for the automated workflow.

### Detailed Workflow

As mentioned above, the program is initialized with the SMILES string for the *cis*-isomer. The *trans* SMILES string is generated automatically by replacing “/N=N*\*” with “/N=N/”. The RDKit package is then used to generate initial three-dimensional coordinates for both the *cis* and *trans* structures.

When systematically constructing the conformers we follow the dihedral angle convention of the ASE package, the *cis* structures are placed at dihedral angles of 10° and 350°, while *trans* structures are initialized at 170° and 190°. These two initial *cis* geometries relax to distinct minima, whereas the two *trans* initializations converge approximately to the same structure. To account for pyrazole rotation, each conformer is duplicated with a 180° rotation of the pyrazole ring, yielding a total of four *cis* and two *trans* conformers for each molecule.

#### Adiabatic Mechanisms

All conformers are then optimized using the L-BFGS algorithm^59^ as implemented in ASE,^60^ with energies and forces provided by the MLIP. Once the minima are obtained and paired correctly, relaxed scans are performed starting from each *cis* geometry to identify approximate transition-state (TS) structures for the three adiabatic mechanisms. Each scan consists of 20 steps. For the two inversion pathways, the scanned coordinate is the corresponding angle α_1_ or α_2_, beginning at the optimized *cis* minimum value and ending at 180°. For the rotational singlet mechanism, the dihedral angle ϕ is scanned from an optimized *cis* structure toward *trans*. In each scan step, the angle is constrained with a steep harmonic potential while all other degrees of freedom relax.

For each mechanism, the structure with the highest energy along the relaxed scan is used as an initial guess for TS optimization with the Sella package.^61^ After optimization, a vibrational analysis is performed to verify that exactly one imaginary frequency exists with a magnitude greater than 100 cm^–1^. The intrinsic reaction coordinate (IRC) functionality in Sella is then used to ensure that the TS connects the correct *cis* and *trans* minima by confirming that one IRC end point has a dihedral angle between 160° and 200° (i.e., *trans*) and the other is either larger than 340° or smaller than 20° (i.e., *cis*). Once validated, Gibbs free energies, evaluated within the RRHO approximation using the Thermochemistry module of the ASE package,^60^ are computed for all *cis* minima and transition states. These values are used together with the appropriate *cis* minimum to compute the activation energies entering eq 1.

We deliberately chose not to employ conformational sampling (e.g., the CREST^62^ approach by Grimme) to search for lower-lying minima or TSs to limit the computational cost. Initial tests on systems for which experimental data are available showed no improvement in accuracy upon including sampling, only a substantial computational overhead.

#### Nonadiabatic Mechanism

The workflow for the triplet-mediated rotational mechanism follows a similar structure as the adiabatic one, but requires sampling both *cis* and *trans* minima. For each of the four *cis* conformers and the corresponding two *trans* conformers, relaxed scans of 20 steps are performed by varying again the central dihedral angle over a total range of 40°. The displaced geometries from these scans serve as starting points for MECP optimization.

MECPs are located by minimizing the energy-difference penalty potential introduced by Bearpark et al.^63^ again using L-BFGS as implemented in ASE. The resulting gradient **g** is computed as follows

9 $$g=-F_{u}+\frac{F_{u}\cdot \Delta F}{\Delta F\cdot \Delta F}\Delta F-2\left(E_{u}-E_{l}\right)\frac{\Delta F}{\left|\Delta F\right|}$$

with the indices on forces **F** and energies *E* indicating either the lower (l) or upper (u) state.

Two MECPs are typically found for each combination of *cis* and *trans* minima, corresponding to the forward and backward directions of rotation. After MECP optimization, Gibbs free energies are computed, and forces on both the singlet and triplet surfaces are evaluated. Using the MECP geometries and the RRHO free energies, the nonadiabatic rotational rate is computed according to eq 7. The spin–orbit coupling (*H*_SO_) required for the transmission coefficient is set to a constant value of 50 cm^–1^, based on our observation that SOC values at the MECPs cluster tightly around this value (see Section S4). The predicted nonadiabatic rate is only weakly sensitive to this value: in the weak-coupling regime relevant here, eqs 2–4 give a transmission coefficient ϵ ∝ *H*_SO_^2^, so that *k*_R_S/T__ ∝ *H*_SO_^2^. The full spread of SOC values we sampled (Section S4) therefore translates into a variation of the triplet-mediated rate of less than a factor of 2, well below the intrinsic accuracy of the workflow. Moreover, R_S/T_ contributes to the total relaxation rate only when it is the dominant channel, further limiting the impact of this approximation on the reported rates. All computed energies, free energies, and rate constants for each compound are written to a JSON file. All optimized structures (minima, TSs, and MECPs) are collected in xyz-file format. SMILES, rates, and vertical excitation energies are also collected in a CSV table for further analysis.

## Supplementary Material



## Data Availability

All predicted rates, associated structures, workflow scripts, and trained potentials are available on Zenodo (https://zenodo.org/records/19249873).

## Notes

Details on (i) the performance of the employed MLIP on the test set, (ii) the distribution of vertical excitation energies of the *trans*-conformer, (iii) a full list of model and training hyperparameters, and (iv) spin–orbit coupling calculations (PDF)
