# Supplementary material for: Design Rules for Tuning Thermal Lifetimes of Arylazopyrazole Photoswitches
Source: ACS Cent Sci. 2026 Aug 4;12(8):1246–57. doi: 10.1021/acscentsci.6c00586 (PMC13523503; doi:10.1021/acscentsci.6c00586)
Supplement: Supplementary file 1 [file oc6c00586_si_001.pdf]

Supporting Information: Design Rules for Tuning Thermal Lifetimes of Arylazopyrazole Photoswitches

Maximilian X. Tiefenbacher,[†,‡,§] Johannes C. B. Dietschreit,[*,†,‡] Simon Axelrod,[¶] Rafael Gómez-Bombarelli,[¶] and Leticia González[*,†,‡]

†*Institute of Theoretical Chemistry, Faculty of Chemistry, University of Vienna, Währinger Straße 17, 1090 Vienna, Austria.*

‡*Research Platform on Accelerating Photoreaction Discovery (ViRAPID), University of Vienna, Währinger Straße 17, 1090 Vienna, Austria.*

¶*Department of Materials Science and Engeniering, Massachusetts Institute of Technology, 77 Massachusetts Ave. Cambridge, MA 02139*

§*Vienna Doctoral School in Chemistry, University of Vienna, Währinger Straße 42, 1090 Vienna, Austria.*

E-mail: johannes.dietschreit@univie.ac.at; leticia.gonzalez@univie.ac.at

Contents

1 Training errors **S3**

2 Excitation energies **S6**

3 Training details **S7**

4 Spin orbit coupling calculations **S9**

References **S11**

1 Training errors

Figure S1 presents the performance of the final model on the test set in the form of parity plots for all targeted adiabatic states and their corresponding gradients. Each subplot reports the mean absolute error (MAE) and root mean squared error (RMSE), given in kcal/mol for energies and kcal/(mol Å) for gradients, as well as the coefficient of determination (R^2) and the Spearman rank correlation coefficient (ρ).

Both R^2 and ρ indicate excellent agreement between predictions and reference data across all states. The MAE remains below 1 for all quantities, whereas the RMSE is only below 1 kcal/mol for the ground-state energy. The noticeable difference between MAE and RMSE suggests the presence of a small number of outliers that disproportionately affect the RMSE. These points are not visible in the plots, likely because the axes span several orders of magnitude.

While these test-set metrics characterize the accuracy of the model on single-point predictions, they do not directly assess its reliability for the downstream tasks of geometry optimization and Gibbs free energy evaluation, where errors in the predicted potential energy surface can accumulate over the course of an iterative optimization. To probe this directly, we performed independent NN- and QM-level (BHHLYP-D3(BJ)/def2-SVP) geometry optimizations of all *cis* and *trans* minima for two representative AAP derivatives spanning different substitution patterns, and compared the resulting structures and free energies (Table S1).

The NN- and QM-optimized geometries agree closely, with RMSD values between 0.004 and 0.068 Å across all seven converged minima, indicating that the NN locates essentially the same stationary points as the reference method. We note that for one *cis* minimum of the second, more sterically complex derivative, the QM optimization did not converge, so no direct comparison is available for that point. Across all converged minima, $\Delta G_{\mathrm{NN-QM}}$ is consistently positive, *i.e.*, the NN systematically overestimates the absolute Gibbs free energy relative to the QM reference. Because this offset is consistent in sign and similar in magnitude

Table S1: Comparison of optimization performance between the trained neural-network potential (NN) and the reference electronic structure method (BHHLYP-D3(BJ)/def2-SVP, QM) for two representative AAP derivatives. For each optimized minimum, we report the structural deviation (RMSD, Å) between the NN- and QM-optimized geometries, and the difference in Gibbs free energy ($\Delta G_{\mathrm{NN-QM}} = G_{\mathrm{NN}} - G_{\mathrm{QM}}$, kcal/mol; electronic energy plus vibronic correction) evaluated at each respective optimum.

minimum	$\mathrm{RMSD}_{\mathrm{NN-QM}}$ [Å]	$\Delta G_{\mathrm{NN-QM}}$ [kcal/mol]
**para NH_2 3Me**		
cis 1	0.029	1.82
cis 2	0.052	3.01
trans 1	0.052	1.26
trans 2	0.005	2.86
**ortho CF_3 ortho' $N(Et)_2$ 2Me**		
cis 1	0.004	3.53
trans 1	0.036	2.68
trans 2	0.068	3.67

across both *cis* and *trans* minima and across both derivatives, it largely cancels when free energy *differences* rather than absolute values are considered, as is the case throughout our screening. We therefore consider the present level of agreement, both structural and energetic, sufficient to support the conclusions drawn from the large-scale screening.

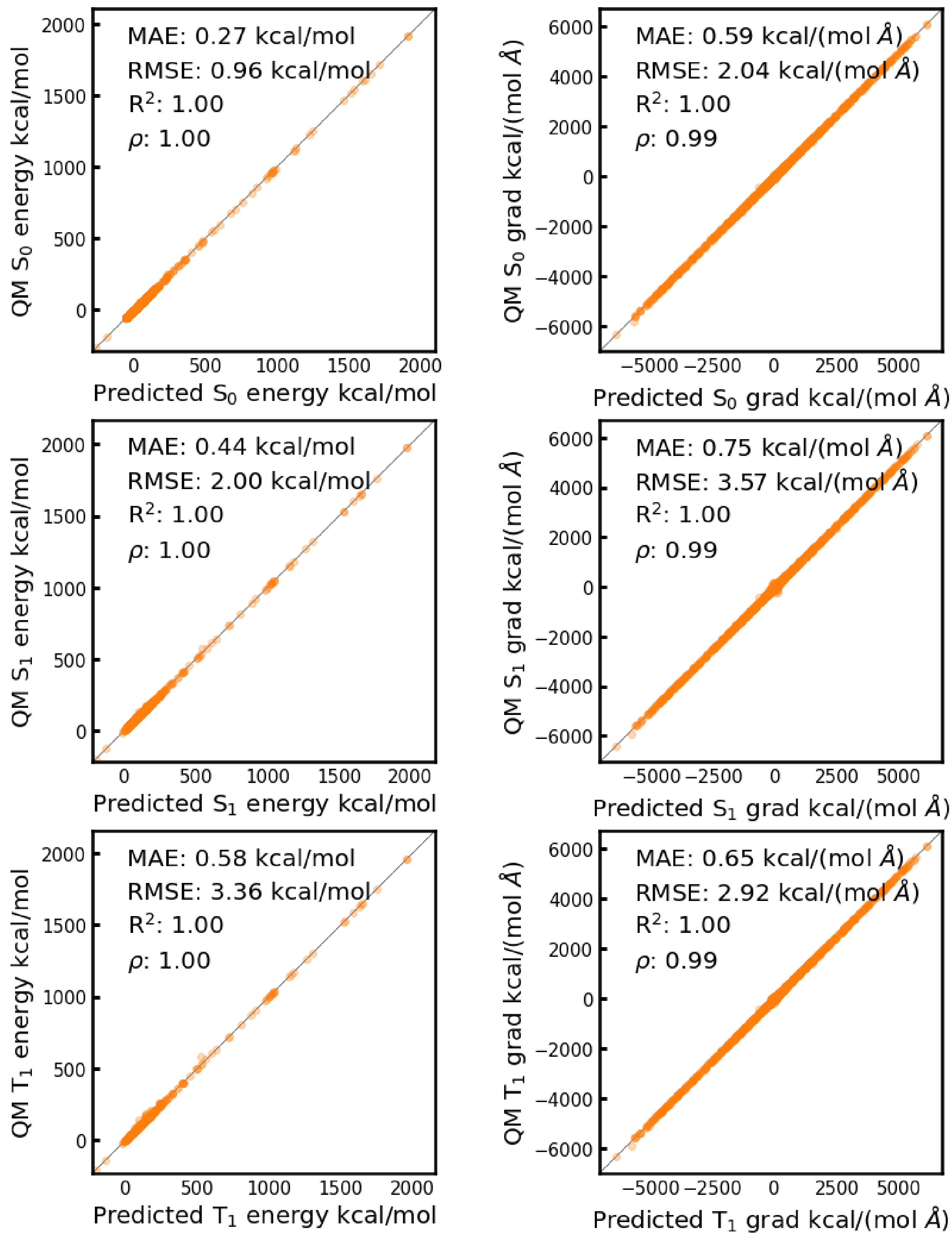

Figure S1: The left side contrasts the predicted energies with the quantum mechanical ground truth for the S_0,S_1, and T_1 states. The right side does the same for all elements of the gradient vectors for the same states. Each subplot also includes the mean absolute error (MAE), the root mean squared error (RMSE), coefficient of determination R^2 and the spearman coefficient (ρ).

2 Excitation energies

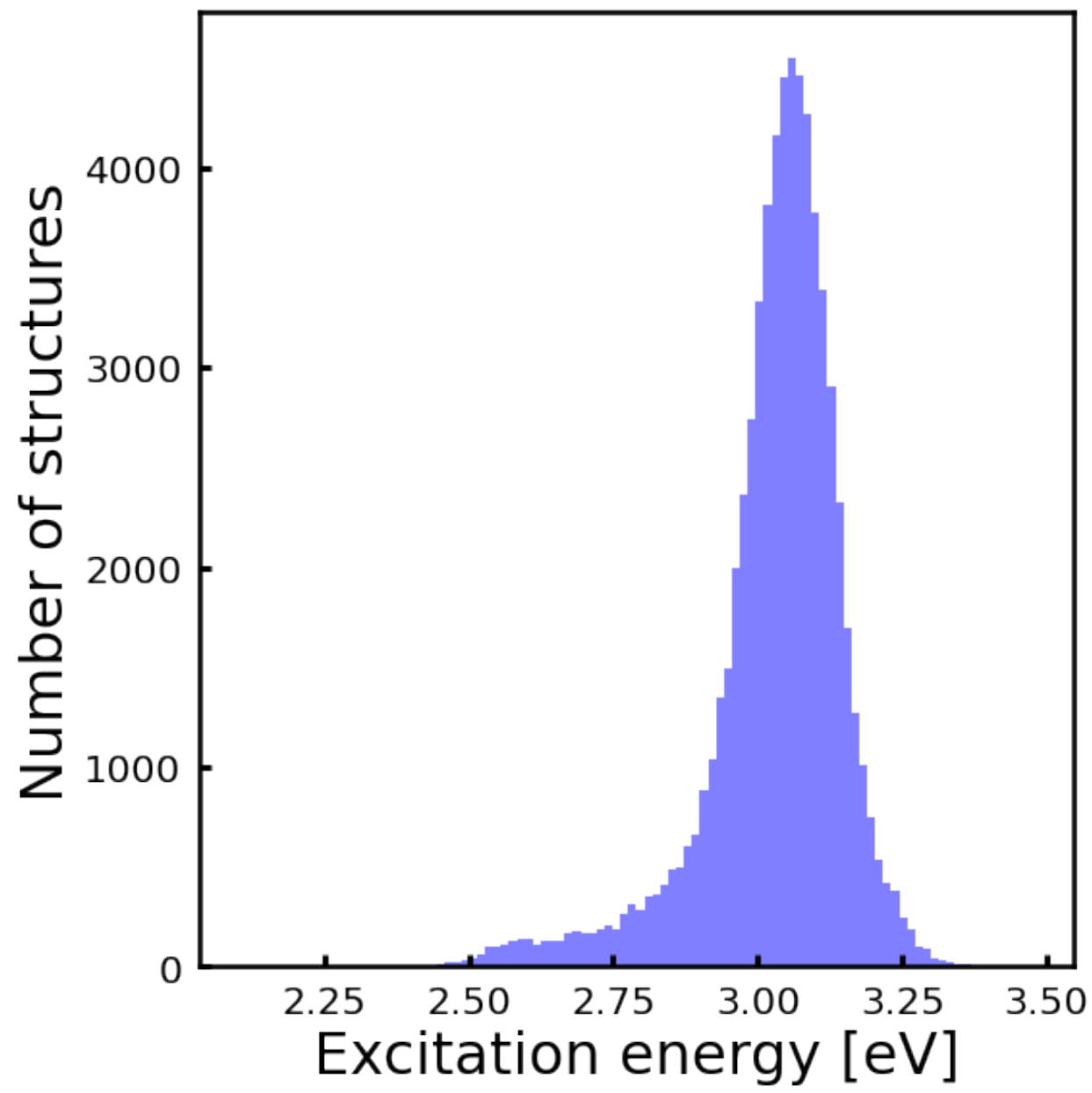

Figure S2: The excitation energies in eV for all trans minima are shown in a histogram.

Figure S2 shows the vertical excitation energies from the electronic ground state S_0 to S_1 for all *trans* minima, computed as energy differences between the corresponding predicted states. The structures were generated using RDKit and subsequently optimized with the trained MLIP, as described in the main text.

The predicted excitation energies predominantly cluster around 3 eV, with the lowest values approaching approximately 2.5 eV. These excitation energies remain at least 0.5 eV above the near-infrared (therapeutic) window, where light penetration in biological tissue is deepest.

3 Training details

The neural network architecture used is the `PAINN_dispersion` model from the `nff` package available on github via `https://github.com/learningmatter-mit/NeuralForceField`. We chose a radial cut off radius of 5 Ångström for the message passing with 32 gaussian functions and 20 radial basis functions. There are three message passing layers in total with a feature length of 256. The energies of the three different states are predicted by a feed forward neural network after the message passing. Each of them has two hidden layers of sizes 256 and 128, leading to one output neuron for the energy of the corresponding state. The model adds the D3 correction[S1] with Becke-Johnson damping on top of the energy prediction to capture long term interactions. Table S2 summarizes all hyperparameters.

Table S2: A list of hyperparameters and their settings, which are related to the architecture of the neural network.

activation function	swish
cutoff	5 Ångström
Number of gaussians	32
Number of radial basis functions	20
feature dimension	256
number of convolutions	3
dispersion type	D3 bj[S1]

The training setup and execution were carried out using in-house software. The data set was partitioned randomly into training, validation, and test sets in an 80:10:10 ratio. The hyperparameters employed for all neural networks are summarized in Table S3. A batch size of 32 was used throughout training. For a single sample, the loss function was defined as

$$\mathbb{L} = \frac{1}{n_{\text{states}}} \left[0.2 \sum_{i=0}^{n_{\text{states}}} \left| E_i^{\text{pred}} - E_i^{\text{QM}} \right| + \frac{1}{3n_{\text{atoms}}} \sum_{i=0}^{n_{\text{states}}} \sum_{n=0}^{n_{\text{atoms}}} \sum_{j=0}^{3} \left| F_{i,n,j}^{\text{pred}} - F_{i,n,j}^{\text{QM}} \right| \right], \tag{S1}$$

where n_{states} denotes the number of electronic states and E_i^{pred} and E_i^{QM} are the predicted and quantum mechanical reference energies, respectively. The force components $F_{i,n,j}$ correspond to atom n in electronic state i, with $j \in \{x, y, z\}$ indicating the Cartesian component. The

total force error is normalized by the number of atoms n_{atoms} in the molecule. By scaling the energy term with a factor of 0.2, the force contribution is effectively weighted five times higher than the energy contribution. Energies are expressed in kcal/mol and gradients in kcal/(mol Å).

The initial learning rate was set to 10^{-4} and reduced by a factor of 0.5 if the validation error did not decrease for 25 consecutive epochs. Training was terminated once the learning rate reached 10^{-7} or after a maximum of 1000 epochs.

Table S3: The most important hyperparameters from the neural network.

batch size	32
loss function	mean absolute error
weight for energies in loss	0.2
weigh for forces in loss	1
initial learning rate	10^{-4}
learning rate decay	0.5
minimal learning rate	10^{-7}
learning rate patience	25
maximum epochs	1000

To avoid systematic energy differences between molecules of varying sizes, a linear regression was performed to determine an average per-element reference energy. This offset was subtracted from each molecule prior to training and added back to all predicted energies during inference. In our Zenodo archive we provide the trained model and the training set.

4 Spin orbit coupling calculations

To validate the use of a constant spin-orbit coupling (SOC) value in the rate model, we computed SOCs at minimum-energy crossing points (MECPs) for nine singly-substituted 3Me AAP derivatives, spanning the *ortho*, *meta*, and *para* positions and substituents with both negative and positive Hammett parameters (CN, F, NH_2), together with the unsubstituted parent compound. The MECPs were found using the algorithm described in the Methods section of the main text, which is briefly repeated here.

Relaxed scans are performed starting either from the *cis* or *trans* minima along the central dihedral angle ϕ around the azo bond. Each of these scans creates 20 equidistant structures, with the final structure being rotated by 40° with respect to the starting minimum. From each scan, the final structure is used as a starting point for the MECP optimization following the gradient defined in Eq. (8) of the main paper using the L-BFGS algorithm as implemented in ASE. The optimized MECPs were subsequently used as input for quantum mechanical calculations with QChem version 5.4 at the same level of theory as was used to generate the training set, *i.e.* SF-TDDFT with BHHLYP-D3(BJ)/def2-SVP. The resulting SOCs are shown in Table S4. The large majority of values fall within a narrow range of approximately 48–52 cm^{-1}, irrespective of substituent identity, ring position, or Hammett parameter sign. The notable exceptions are three MECPs for the *ortho*-NH_2 derivative, where the relevant triplet state switches character from T_1 to T_2 (marked with * in Table S4). As the Hammett parameters have previously been shown to have a strong correlation with the thermal relaxation rate for these specific AAPs,[S2] the approximation of a constant SOC value for the dominant T_1-mediated channel seems valid.

Table S4: Spin-orbit couplings (SOCs, in cm^{-1}) computed with the reference method at the ML-predicted minimum-energy crossing points (MECPs) for singly-substituted 3Me AAP derivatives, with the aryl substituent in the *ortho*, *meta*, or *para* position. Substituents span both negative (NH_2, F) and positive (CN) Hammett parameters, together with the unsubstituted parent compound. Values are computed between S_0 and the lowest triplet state of T_1 character; values marked with * instead correspond to the S_0-T_2 coupling, as the relevant triplet state switches character from T_1 to T_2 at that MECP. Missing entries (–) indicate substituent/position combinations or MECPs for which no converged crossing point was obtained.

Substituent	ortho	meta	para
CN	49.7834	51.1244	51.3256
CN	49.9578	51.4579	51.2260
CN	51.1059	51.6919	50.5592
CN	49.9349	51.3550	51.1899
CN	51.9583	49.4759	48.9977
CN	49.9559	51.7873	51.1880
CN	49.7453	51.2499	49.0673
CN	49.9528	–	51.2268
F	49.5651	51.1017	49.7389
F	49.5010	50.2280	48.8488
F	51.1048	51.6440	50.5047
F	49.7322	51.6929	49.7372
F	51.0577	51.9096	48.3193
F	49.8151	51.5747	49.6793
F	–	51.9800	50.3667
F	–	51.5941	49.7039
NH_2	47.0690	51.0388	47.3084
NH_2	66.9877*	49.8367	46.8667
NH_2	43.4648*	51.6172	46.9183
NH_2	66.9878*	51.4320	46.1598
NH_2	43.0109	29.0825	48.0233
NH_2	66.9887*	51.7459	46.8431
NH_2	–	–	–
NH_2	–	–	–
No substitution	–	51.4319	–
No substitution	–	51.1023	–
No substitution	–	50.7318	–
No substitution	–	50.0400	–
No substitution	–	51.3014	–
No substitution	–	50.6274	–
No substitution	–	49.4021	–
No substitution	–	50.6274	–

References

(S1) Grimme, S.; Ehrlich, S.; Goerigk, L. Effect of the damping function in dispersion corrected density functional theory. *Journal of Computational Chemistry* **2011**, *32*, 1456–1465.

(S2) Schlögl, K.; Singer, N. K.; Dreier, D.; Kalaus, H.; Conceição, R. C. O.; Mihovilovic, M. D.; González, L. Mechanistic Insight into Para-Substituent Control of Thermal Half-Lives in Arylazopyrazole Photoswitches. *Angewandte Chemie International Edition* **2025**, *64*, e202514433.
